# Real-Time Exposure to Alcohol Content in Digital Media in Adolescents: Protocol for a Multiburst Ecological Momentary Assessment Study

**DOI:** 10.2196/50649

**Published:** 2025-09-08

**Authors:** Kristina Jackson, Joy Gabrielli, Suzanne Colby, Tyler Wray, Tim Janssen, Michelle Rogers, Cassandra Delapaix

**Affiliations:** 1 Rutgers Addiction Research Center (RARC) Department of Psychiatry Rutgers Robert Wood Johnson Medical School Piscataway, NJ United States; 2 Department of Clinical and Health Psychology University of Florida Gainesville, FL United States; 3 Center for Alcohol & Addiction Studies School of Public Health Brown University Providence, RI United States; 4 Department of Behavioral and Social Sciences Brown University Providence, RI United States

**Keywords:** media, entertainment, social, exposure, adolescent, alcohol use, ecological momentary assessment, passive

## Abstract

**Background:**

Digital media frequently contains positive portrayals of alcohol content, which has been shown to be associated with alcohol-related cognitions and behaviors. Because youth are heavy media consumers and have access to unsupervised, repeat viewing of media content on their personal mobile devices, it is critical to understand the frequency of encountering alcohol content in adolescents’ daily lives and how adolescents engage with the content.

**Objective:**

This paper outlines the study protocol for examining adolescents’ exposure to alcohol-related content in digital media within their natural environments.

**Methods:**

Adolescents (N=302; 31.8% boys, 16.2% nonbinary, 51.3% girls; 25.8% Asian, 3.6% American Indian, 21.5% Black, 4.6% other, 52% White, 25.8% Hispanic or Latinx; mean age 16.21, SD 0.77 y) enrolled in high school were recruited through social media to participate in a prospective study involving bursts of ecological momentary assessment (EMA) reports coupled with longer surveys. We conducted group orientation sessions via videoconference and online surveys, followed by a 21-day EMA period that included scheduled reports across 4 daily time blocks, as well as self-initiated reports on media exposure. Reports of alcohol content exposure included details about the platform, level of engagement, source characteristics, beliefs and perceived norms about the content, the viewing context, and whether the content was sponsored or branded. The participants submitted exposures to alcohol content as an image (screenshot or photo) or text description to be objectively coded. The participants completed a weekly online survey assessing alcohol use and related cognitions. EMA reports will be merged with coded image and text entries and with data from baseline, weekly, and follow-up surveys. Self-reported alcohol exposure will be explored descriptively, and differences in exposure tested across subgroups. Event-level data will be compared with random prompt data to examine differences at times of exposure versus nonexposure. Prospective associations between media alcohol content exposure and alcohol use will be explored over 1-week and 4-month time frames. Mediation of the association between media alcohol exposure and drinking will be tested to explore putative mechanisms.

**Results:**

EMA data collection took place from February 2022 to August 2023. Data management and preliminary analysis are ongoing. Preliminary data were disseminated through conference presentations in 2024-2025 and manuscripts are ongoing with full results anticipated to be published in 2025-2026.

**Conclusions:**

By characterizing adolescents’ real-world exposure to alcohol content in the media, the study provides critical information to develop and implement interventions to target youth behavior that are well suited to delivery via mobile devices. Next steps are to conduct focus groups to understand participants’ lived experience of exposure to media alcohol content and reactions to proposed intervention targets. This study and subsequent qualitative work will launch a program of research to counter the effects of alcohol-related media exposure as experienced by adolescents in an effort to minimize underage alcohol involvement.

**International Registered Report Identifier (IRRID):**

DERR1-10.2196/50649

## Introduction

### Background

Initiation of alcohol use typically occurs well before the legal drinking age [1-3], which is concerning as early use is associated with short- and long-term adverse outcomes, including acute and prolonged neurobiological impairments [4-6]. The importance of the environment in shaping early drinking behavior is increasingly recognized, with adolescents highly susceptible to the socializing influences of peers and prevailing generational norms [7-10]. Media represents one particularly important contextual influence for adolescents. Youth are heavy media consumers [11], and personal mobile devices such as smartphones and tablets offer youth ease of unsupervised, repeat viewing of personalized media content.

Digital media, which encompasses social media, new media, and entertainment media such as television, movies, and music, frequently contains positive portrayals of alcohol content. Entertainment media features images of and references to alcohol, associating alcohol use with social, sexual, and financial success, with little depiction of the hazards of drinking [12,13]. The digital media environment has rapidly evolved over the past 15 years, and alcohol content is pervasive on social media and YouTube. These platforms are highly interactive, allowing for user engagement through exchange and manipulation of information [14-18]. Youth self-report high exposure to alcohol content in digital media [19-21]. Notably, rigorous review studies have concluded that exposure to media alcohol content is consistently associated with increased risk of early initiation and progression of underage drinking [22-24]. Etiological research on the mechanisms underlying the association between media exposure and drinking points to cognitive and social mechanisms, including perceived norms [25,26], cognitions (expectancies [27,28] and drinker prototypes [29]), identity [30], and attitudes (favorability [31] and evaluative conditioning [32,33]).

Despite this etiological research, virtually no information is available about in vivo exposure to media alcohol content. Understanding the frequency of encountering alcohol content in adolescents’ daily lives and how adolescents engage with the content is critical for efforts to reduce exposure. Determining how such exposure influences a person is important for mitigating the impact of the exposure. This study seeks to understand when, where, and how much teenagers are exposed to alcohol content in digital media and its influence on attitudes, cognitions, and behavior. Study outcomes will address gaps in the literature, focusing on several limitations in earlier research: the focus on a single aspect of media exposure (eg, social media), assessment of primarily passive exposures through screen media, use of primarily retrospective self-reports of media exposure and behavior, and coarse time frames of assessment.

Our study focuses on older adolescents. Younger teenagers tend to have little experience with alcohol, report low intention to drink, and perceive negative consequences of drinking, with a shift to positive beliefs occurring toward later adolescence [34,35]. The link between media exposure and social norms also may be tenuous at younger ages, when fewer youth have friends or same-age peers who consume alcohol. Thus, there may be limited variability in drinking cognitions and peer norms in young adolescents, as documented in our own work [36,37]. Older adolescents may be well suited to an intervention that provides tools for countering effects of exposure to media alcohol content, as content may be more relevant, perceived norms and cognitions may be more modifiable, skills can be practiced as alcohol is available and consumed, and this age accesses new media continually and with little supervision.

The first aim of the project is to quantify and characterize in vivo exposure to alcohol content in digital media, including entertainment media (film, television, and popular music), social media, and YouTube, in terms of frequency and duration, medium and format, and context. The second aim is to examine prospective associations between exposure to alcohol media content and alcohol involvement (willingness, use, heavy use, and problems) across short- and longer-term time frames. Aim 3 identifies putative mechanisms of these associations, including perceived norms, cognition (expectancies and drinker prototypes), and identity (personal and social). The study’s multiple-burst longitudinal design will permit direct comparison of cognitions, norms, and context at moments of exposure versus nonexposure to media alcohol content and will provide important information regarding timing and directionality (or bidirectionality) of influence between exposure to media alcohol content and youth alcohol use.

### Objectives

This paper describes the protocol for a study that examines exposure to digital media alcohol content in the natural environment among adolescents (aged between 15 and 18 years). The project involves 3 bursts of ecological momentary assessment (EMA) reports coupled with longer surveys of broader content. The participants were also asked to submit exposures to alcohol content in the media in the form of an image (eg, screenshot and photo) or text description to be objectively coded. The study is the first in vivo, EMA investigation of exposure to alcohol content in the media that considers a wide array of entertainment and digital media.

## Methods

### Design

**Figure 1:**
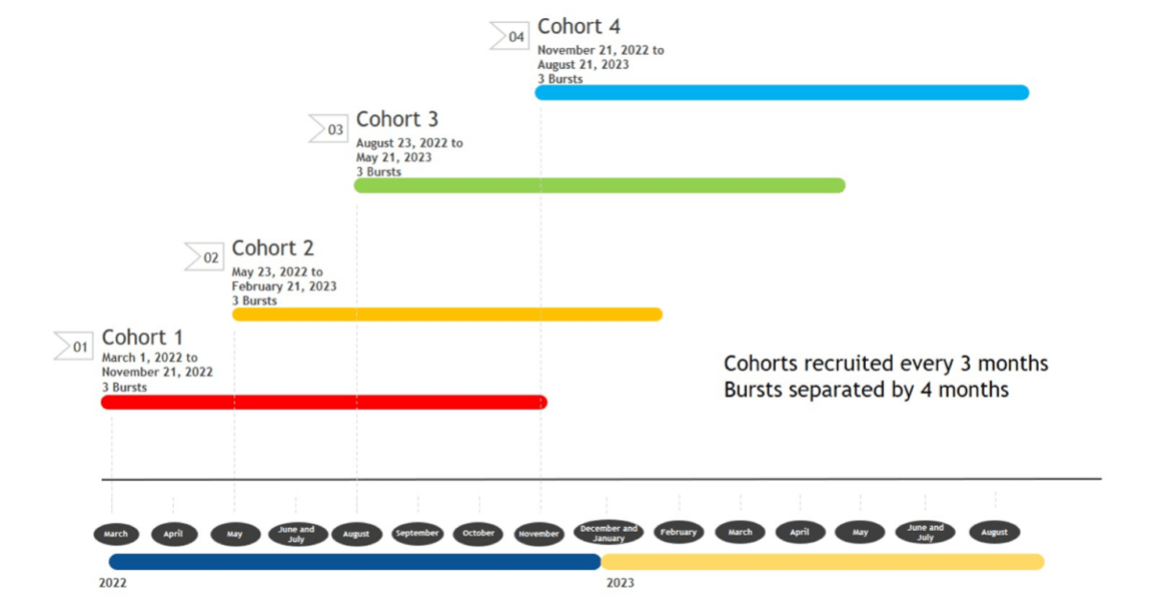
Multiple cohort multiburst study design to examine exposure to alcohol content in the media among adolescents in the United States.

Adolescents (N=302) currently enrolled in high school were recruited through social media to participate in an EMA study with three 21-day bursts of daily EMA assessments separated by 4-month intervals (Figure 1). Following a baseline session, EMA reports were coupled with weekly 7-day timeline follow-back (TLFB) assessments during the bursts.

**Figure 2:**
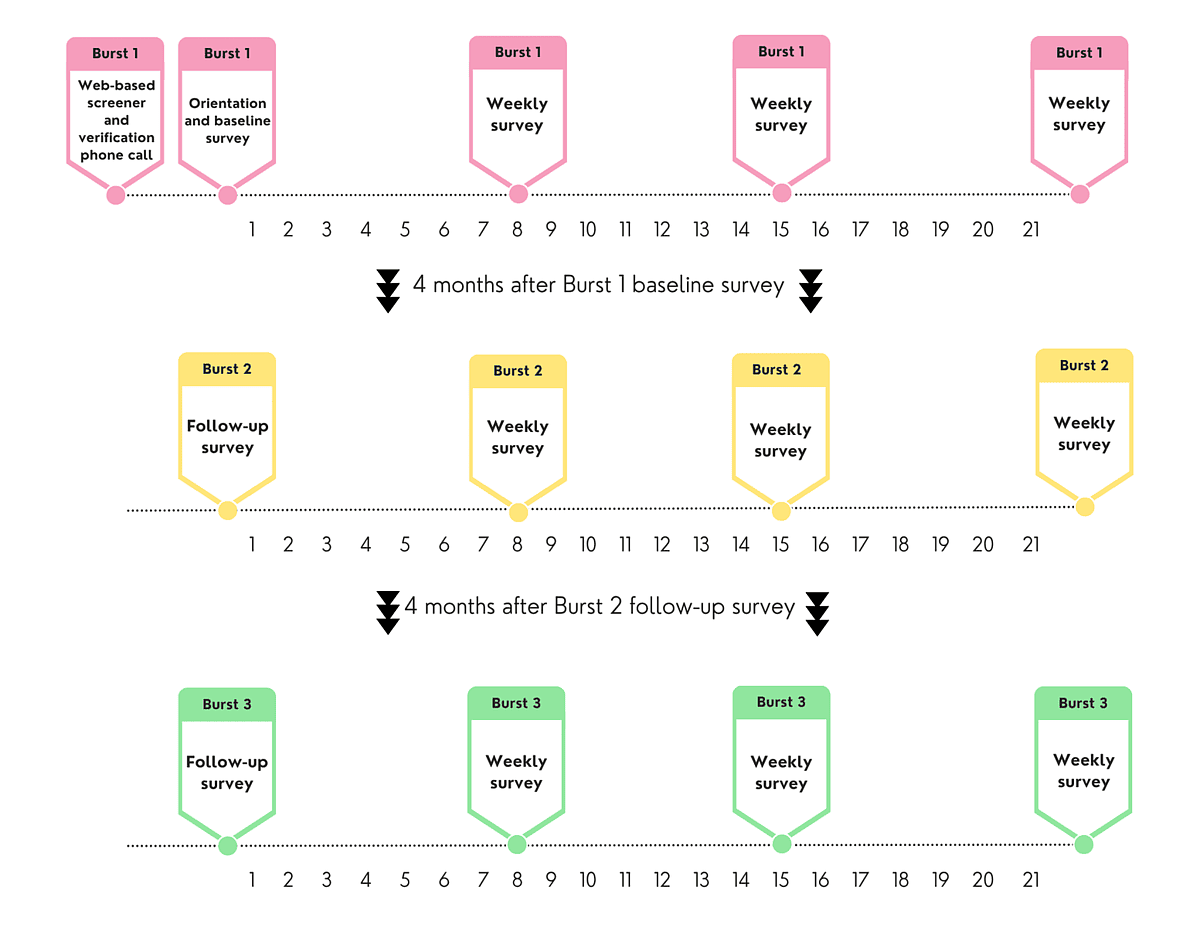
Visual timeline of study procedures.

In addition, 2 follow-up surveys were administered preceding each burst (Figure 2). The measurement burst design [38,39] combines a longitudinal panel design with experience sampling methods and permits investigation of processes in both short term and long term.

The participants completed a group orientation session conducted over Zoom (Zoom Communications, Inc) where study procedures were explained and the study smartphone app was installed. Before the orientation, the participants completed self-report measures in Qualtrics (Qualtrics International Inc). EMA reporting began for each cohort of participants from 1 to 12 days following their orientation session. Each day during the study, the participants used the study smartphone app to report on exposure to alcohol content in the media and responded to a series of items assessing type of media, reaction to the content, and attitudes and cognitions about the content. The participants also used the app to submit images or text descriptions of the content, which were subsequently content coded by the research team.

### Youth Advisory Board

We formed a youth advisory board (YAB) of high school students in the southern New England area to obtain youth perspectives to (1) understand adolescent experiences encountering alcohol content in the media and (2) obtain specific input on study methods to make our study relevant and engaging to present-day high school youth. We recruited youth who could share and reflect the diverse perspectives of the community by involving students with variation in age, gender, racial and ethnic group, school, and region. The YAB contributed to the development of our recruitment and retention strategies and provided input on the study protocol and assessment battery. The YAB members received an annual honorarium of US $100, distributed in 2 payments of US $50 every 6 months. We informed members that they had to attend at least 4 of 6 meetings to receive the full honorarium. From the application process, it seemed that most members also had an intrinsic interest in belonging to the group and offering their perspectives. Further information about the YAB and associated methods will be presented in a separate forthcoming paper.

### Participants

We recruited 302 eligible participants into 4 cohorts enrolled in the winter, spring, summer, and fall of 2022. Splitting up enrollment into multiple cohorts enhanced feasibility by reducing burden on research staff. It also permits examination of seasonality separately from testing effects. We will attend to seasonality as we anticipated potential seasonal differences in response rates, alcohol use, and access to media and electronic devices. Although there is a paucity of literature examining seasonal patterns in adolescent compliance with daily surveys, we expected that response rates might differ over summer months as compared to the school-year assessments. For some youth, response rates might be expected to be higher during the summer due to fewer responsibilities and a less structured environment; for others, response rates may be lower due to travel or structured summer activities. We also anticipated that participants would have greater access to social and entertainment media during the summer months, which is a time for relaxation, exploration, and social connection. Finally, the literature indicates seasonal variation in alcohol consumption, at least in college students and adults. Higher rates of alcohol use are seen during times of holidays and summertime [40-43], with 1 study documenting prominent peaks in daily drinking corresponding to summertime and holiday weeks known to be associated with heavy consumption over a 2-year period [44].

Participant inclusion and exclusion criteria included (1) being aged between 15 and 18 years, (2) currently enrolled in high school (9th through 12th grades), (3) able to read simple English, (4) owned a smartphone (iPhone [Apple, Inc] or Android [Google LLC]), and (5) lived in the United States (verified via phone call by a research assistant [RA]). In addition, we only included youth who had consumed alcohol or had at least 1 close friend who drank alcohol, to ensure participants had some familiarity with alcohol while capturing a range of drinking experiences. We limited our sample to adolescents enrolled in high school to ensure similarity in context, as the environment in a high school can vastly differ from the middle school environment or the environment following high school, which spans workforce entry through 4-year college enrollment.

**Figure 3:**
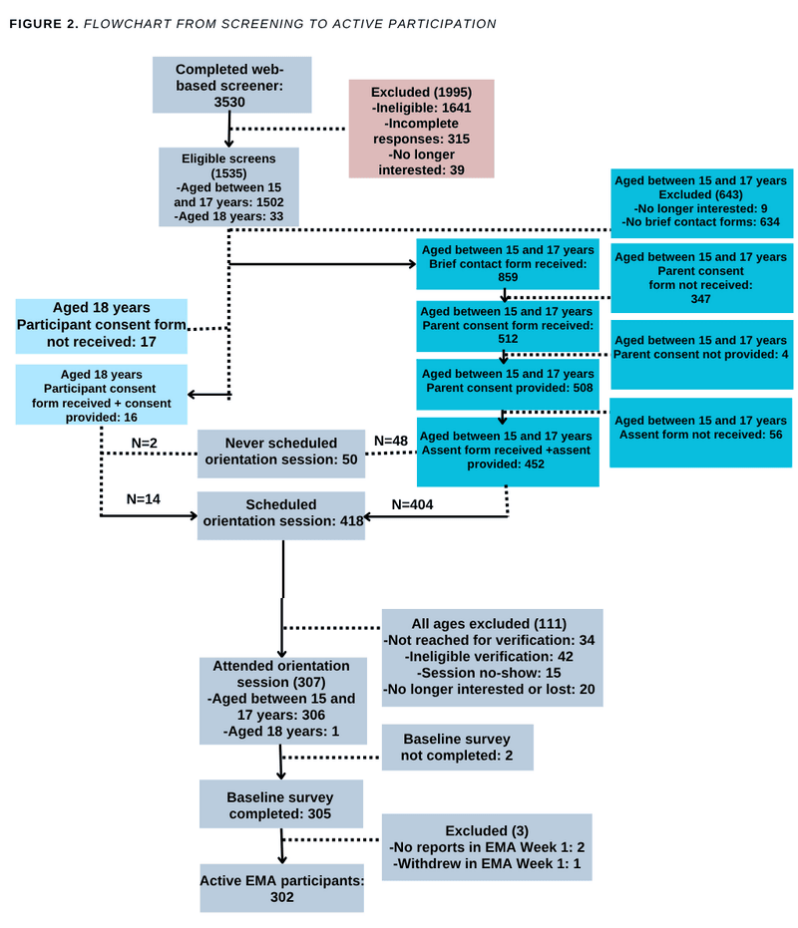
Flow diagram of participant screening and enrollment through active participation. EMA: ecological momentary assessment.

Figure 3 presents a flow diagram of participant screening and enrollment. During the recruitment phase, 3530 people completed our screening questionnaire on the online survey platform Qualtrics. Of those, 1535 (43.5%) participants were eligible to continue the enrollment process, including 16 of 33 (48%) participants who were aged exactly 18 years and who completed the consent form. The remainder of eligible participants were younger than 18 years and were asked to complete a brief contact form (859/1502, 57.2% respondents), following which parental permission was sought. A total of 512 parental consent forms were returned, with 508 parents agreeing to their child’s participation; of these, 452 (89%) also returned assent forms to participate. The students lost following the assent or consent completion were either nonresponsive to our subsequent attempts to reach them by phone to confirm orientation or were withdrawn because we could not verify their identities. Ultimately, 305 students enrolled in the study, meaning they provided consent or assent, attended a study orientation, and completed a baseline survey. A total of 3 individuals withdrew themselves from the study at days 1, 6, and 8, respectively, and are not counted in the final analytic sample. The 302 active participants were enrolled in 4 cohorts, 3 months apart (C1: n=56, 18.5% participants; C2: n=101, 33.4% participants; C3: n=103, 34.1% participants; and C4: n=42, 13.9% participants). In the final sample, 63.9% (193/302) of participants reported being assigned female at birth; gender distribution was reported as follows: 4.6% (1/302) nonbinary, 5.3% (16/302) gender nonconforming, 32.7% (99/302) boys (cis- or trans-), 52.6% (159/302) girls (cis- or trans-), and 4.6% (14/302) other. Our first 2 cohorts were predominately composed of participants assigned female at birth, consistent with other contemporaneous research that uses social media platforms for recruitment. For recruitment of the third and fourth cohorts, we sought to enroll more youth who were assigned male at birth through targeted social media advertisements; our final sample was still skewed toward youth who were assigned female at birth but with diversity in gender identity. Mean age was 16.21 (SD 0.77) years, and 1.3% (4/302) were in the 9th grade, 17.2% (52/302) were in the 10th grade, 44.0% (133/302) were in the 11th grade, and 37.4.% (113/302) were in the 12th grade. The sample exhibited racial and ethnic heterogeneity, with 25.8% (78/302) Asian, 3.6% (11/302) American Indian, 21.5% (65/302) Black, 5% (15 /302) other, and 52% (157/302) White; 25.8% (78/302) reported being Hispanic or Latinx, and 74.2% (224/302) reported being non-Hispanic or non-Latinx.

### Procedures

#### Recruitment

**Figure 4:**
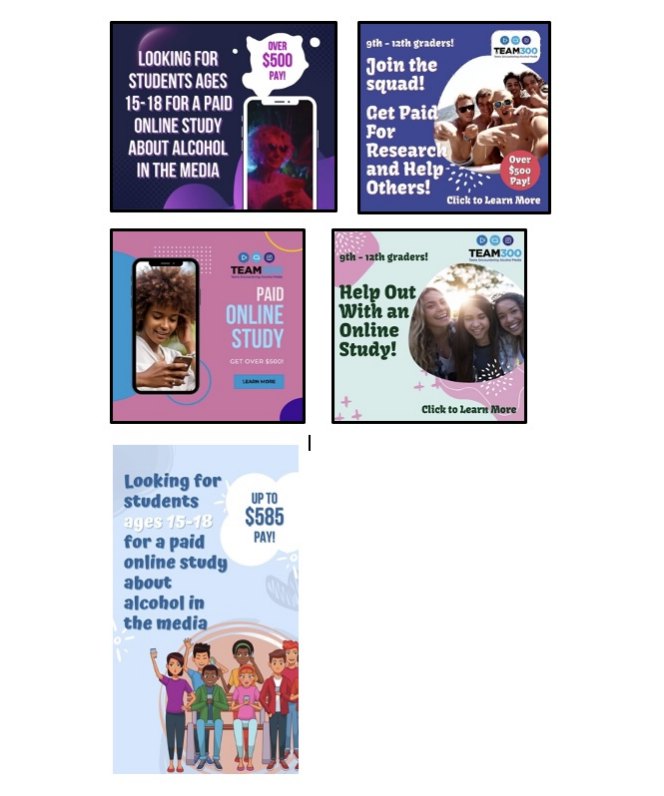
Examples of social media advertisements for recruitment.

We recruited adolescents from social media advertisements, including paid advertisements on Instagram (Meta Platforms, Inc) and Facebook (Meta Platforms, Inc) and posts by research team members on TikTok (ByteDance). Advertisements displayed different combinations of study aspects, including the study name (TEAM300) and logo; engaging phrases (eg, Join the squad!); language about compensation and online format; and relevant images of teenagers, alcohol content in media, and general media use (Figure 4 depicts illustrative advertisements). Advertisements promoted diversity and inclusion by featuring young people from a variety of racial and ethnic backgrounds, with specific visuals and text refined based on feedback from our YAB (refer to the subsequent sections). Study staff ultimately developed advertisements as both still and animated graphics to fit social media feed and story layouts.

Clicking a digital study advertisement directed the individual to a study landing page in Qualtrics that provided basic information about the study, both in brief written and video format, as well as a link to our study website. Those interested in participating were directed to a 14-item anonymous screening survey that assessed basic eligibility criteria. We enabled various security features on Qualtrics (eg, ReCaptcha 2/3 and bot detection) to reduce instances of fraud.

After completing the screening survey, eligible adolescents who were aged 18 years continued directly to the online consent form. Those who consented then provided contact information. Eligible adolescents younger than 18 years provided contact information and subsequently received an email containing study information to be forwarded via email to their parent or legal guardian (hereafter referred to as parents) so that they could provide parental permission. The email contained a link to a Qualtrics landing page for parents, which provided a study overview, an informational video, links to the study website, and a link to the parental consent form. The project website contained a brief description of study procedures and compensation as well as 2 informational videos, 1 geared toward the participants and 1 geared toward parents. The research team, including our staff and YAB members from groups considered historically underrepresented, was featured on our study website. Two frequently asked questions sections were also provided on the website, 1 for adolescents and 1 for parents. The frequently asked questions responded to questions such as “Are there any risks to participating?” “Are you asking me to share anything on my phone or in my social media accounts?” and “What are you asking my child about?” All website materials were written at an 8th-grade reading level or lower. Parents were invited to contact research staff to answer any questions using the study contact information provided, or they could provide online consent for youth participation without additional information needed from project staff. There were no instances of parents reaching out to the team for additional study information before providing parental consent. Once parental permission was obtained within Qualtrics, the participant then received an email with a link to complete their own digital assent form.

Eligible participants who provided consent or assent (with documented parental consent) were directed to an online scheduling platform to select a 45-minute group orientation session. Once scheduled, an RA confirmed the orientation session appointment over the phone. During these calls, staff also confirmed the identities of the participants by asking the participants to confirm their contact information and state the name of their high school, verifying the high school location against the zip code previously given by the participant. We encountered approximately 40 nonlegitimate cases, including issues of contact information not matching previously submitted details, inaccurate high school names or locations, or inability to provide requested information.

#### Participant Orientation and Baseline Assessment

**Figure 5:**
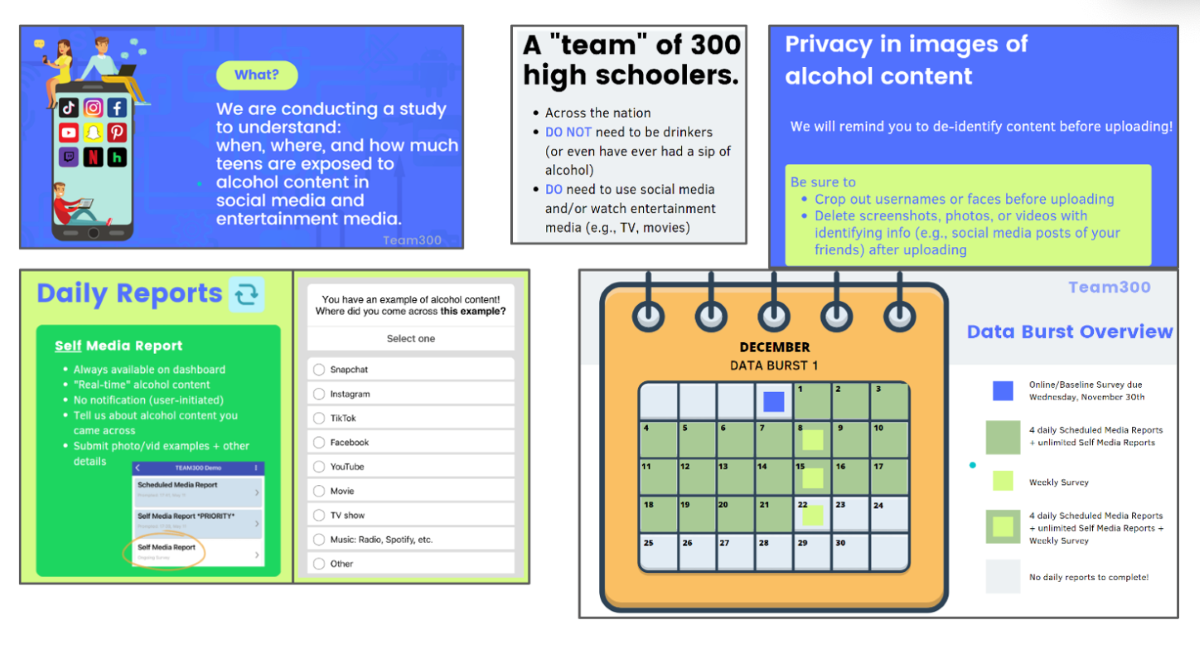
Representative screenshots containing participant instruction guide content.

Orientation sessions were conducted via Zoom in groups ranging from 2 to 10 (median 4) participants. Orientations, which were delivered by RAs, incorporated a slide deck as a visual aid to reinforce study information. Our RAs were diverse across both gender and racial and ethnic groups. The slide layouts, content, and flow of topics were adjusted and refined across time via staff, peers, and YAB feedback, ultimately resulting in standardized orientation session content across cohorts. The presentation included general study elements such as study staff names and position titles, overall study aims, participation timelines, and compensation structure, as well as specific procedural elements such as screenshots of the daily phone app reports and weekly surveys that the participants would complete. Figure 5 shows sample orientation content presented using Canva slides. The goals of the orientation sessions were to promote participant study engagement and teach the participants (1) examples of references to alcohol that might be encountered in day-to-day use of media, (2) the different types of reports, (3) how to submit a self-initiated report about the different types of media content they noticed, (4) how to download and navigate the study app, and (5) how to identify each type of survey and corresponding submission details. The orientation sessions included built-in knowledge checks throughout to ensure that the participants fully comprehended the study protocol.

At the end of orientation, the RA assisted the participants in real time in downloading and logging into the EMA app, MetricWire (MetricWire, Inc), with their study-assigned (nonpersonal) emails and assisted in adjusting phone settings and permissions. Youth were instructed to keep their device with them at all times, except while at school and sleeping, and to ensure they were receiving notifications. They were also provided instructions to adjust notifications in MetricWire (eg, turn off or mute or silence notifications) if desired, such as while sleeping or during sports practice.

**Figure 6:**
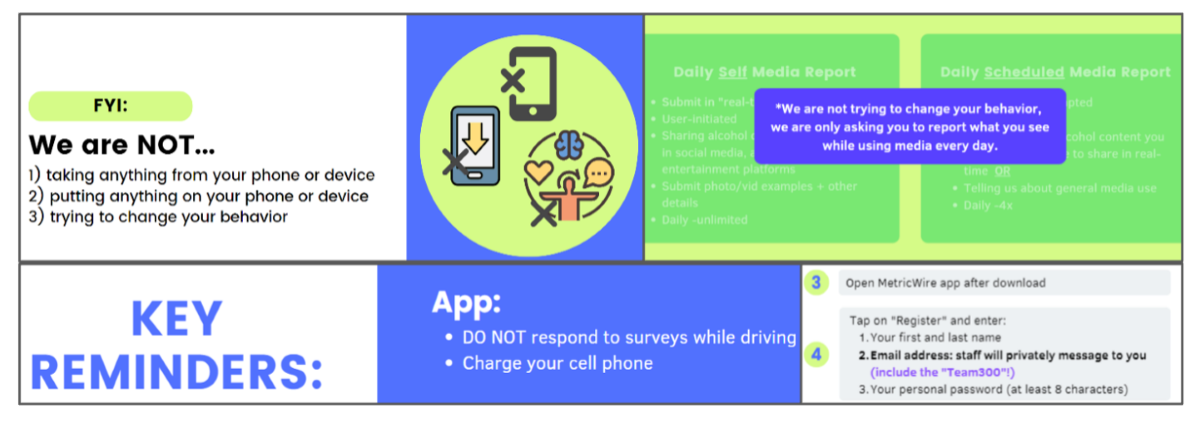
Representative screenshots containing orientation key reminders.

We asked the participants to keep their phone on and charged and not to change phone settings for the duration of the study. The participants were explicitly told that researchers were not taking any information from their phone or device, putting anything on their phone or device, or trying to change their behavior. We emphasized that the participants should not deviate from their normal activities to find alcohol content (Figure 6).

Smartphone use is highly prevalent in this age group and more evenly distributed across race, ethnicity, and household income levels than PCs (resulting in limited bias associated with access) [45]. Thus, we did not anticipate that participants would have any difficulty accessing the survey software. In focus groups conducted before this study [46,47] and in our YAB, we probed device ownership, text plans, and access to Wi-Fi as barriers to participation, and none of the participants indicated this as a concern. It is possible that potentially interested participants were dissuaded from enrolling upon learning that the study procedures involved frequent surveys administered on a smartphone; this might produce a small bias in our sample. We will assess this potential bias using a baseline survey item: “Do you have a data plan for your smartphone?” (response options: yes, unlimited data; yes, but limited data; and no, no data plan), along with indicators of disadvantage such as socioeconomic status, which is measured by free or reduced-cost lunch eligibility and parental education level. It is also worth noting that the digital divide may be less apparent in our study because surveys only require a few minutes of data per day. Following attendance of the orientation session, the participants were emailed a link to the Qualtrics baseline survey. This assessment was requested to be completed before the first day of EMA data collection and included a section where the participants were asked to rate their reasons for joining our study to use the participant’s rationale to personalize our reminder and outreach messages. (For any cohort that did not receive this section in the baseline survey, it was administered instead at their follow-up survey.)

#### Instructions for Reporting Alcohol Content

During orientation, the participants were provided with detailed and illustrated guidance on how to use the study app to report alcohol content that they observed. They were instructed to report each instance of alcohol content in media by initiating a survey continuously available on the study app’s dashboard.

To facilitate coding of alcohol content within the context of a movie, show, video, or song, we instructed the participants to enter a time stamp indicating exactly when the reference to alcohol occurred. When movies, shows, or videos had multiple references to alcohol, the participants were instructed to report on the most noticeable or important scene or example using a free-text entry item that accompanied the event-contingent report. For YouTube, we provided a field for the participants to paste a hyperlink, which could be used to obtain a static image. Finally, the participants were encouraged to submit a comment at the end of the report to describe additional scenes or any other notable information.

For participant reports of alcohol references in images, we similarly asked them to submit these images in event-contingent reports. We taught the participants how to deidentify an image by marking over or cropping out usernames or faces before submitting the image. We recommended that they delete any screenshots or photos with identifying information from their phone or gallery after submitting the report.

**Figure 7:**
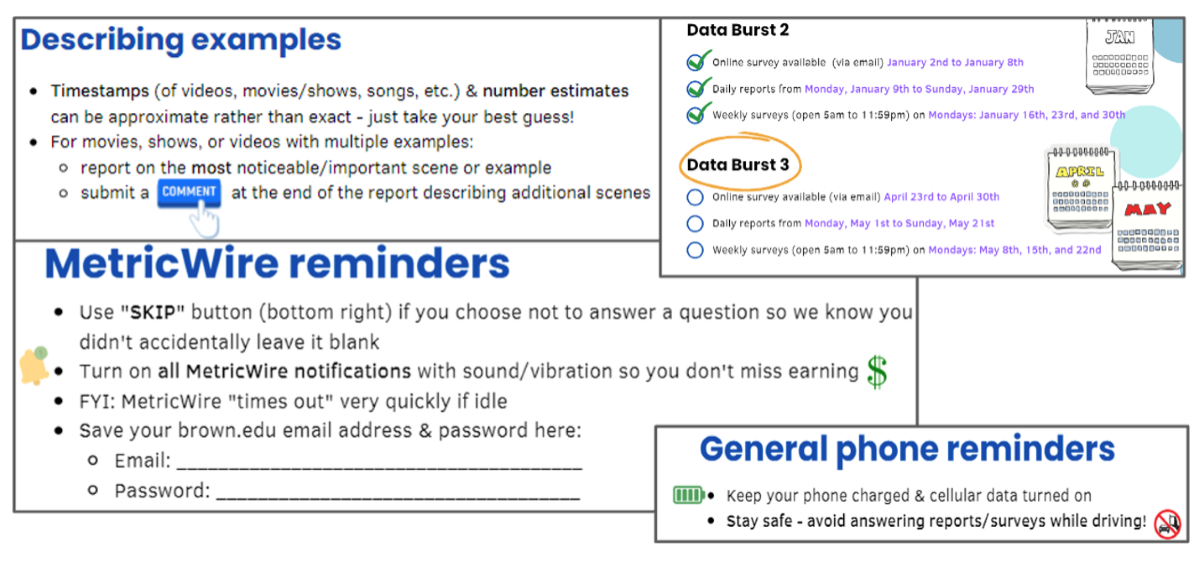
Examples of participant instruction guide content.

Before the start of EMA data collection, staff emailed the participants a participant instruction guide consisting of key reminders about participation rules and expectations (Figure 7). The participants were told that they could refer to this condensed guide at any point for clarification about study terms or survey details. Shortly before each subsequent data burst, staff again emailed the participants the link to the guide, encouraging them to review the information as a refresher of study protocols before starting the EMA portion again.

#### EMA Protocol

**Figure 8:**
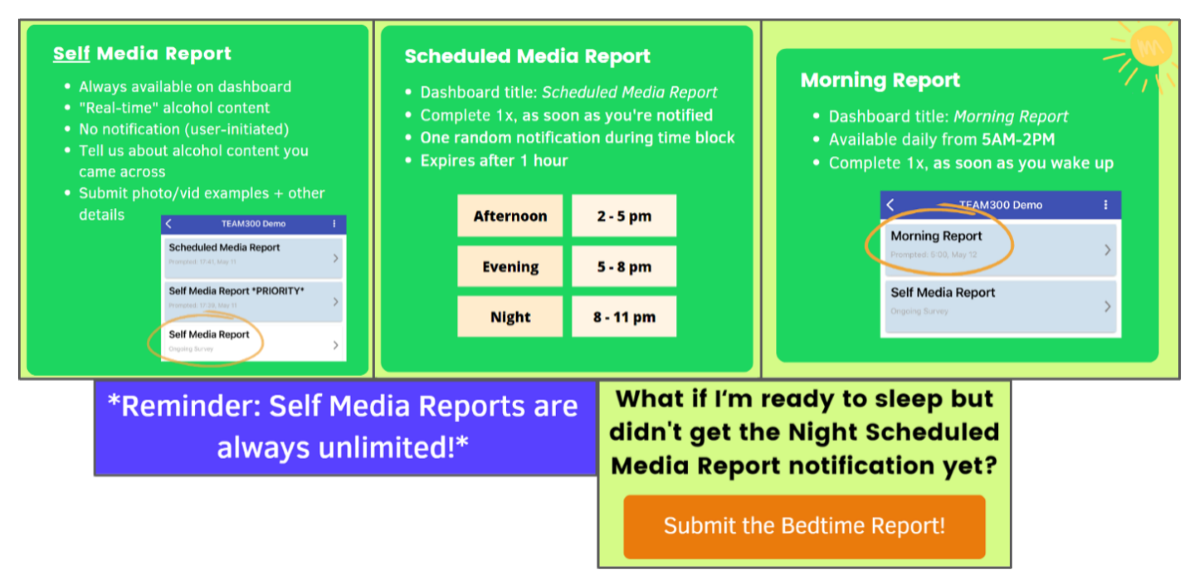
Types of ecological momentary assessment reports, as explained to participants (as provided to participants in the instruction guide).

The first 3-week EMA phase began no more than 12 days after the orientation session. During this monitoring period, the participants completed 2 types of EMA reports on their own personal smartphone devices: scheduled reports (scheduled media reports) and self-initiated reports (self-media reports; Figure 8). The participants were prompted to complete scheduled reports during 4 intervals each day: 5 AM to 2 PM (morning), 2 PM to 5 PM (afternoon), 5 PM to 8 PM (evening), and 8 PM to 11 PM (night). These intervals were chosen to best align with adolescents’ schedules, with the selection of a very early morning survey prompt informed by discussion with members of the YAB. The prompt for the morning report was delivered at 5 AM, and the survey stayed available on the app dashboard until 2 PM each day, with reminders issued at 8 AM and 12 PM if necessary. The participants were prompted to complete afternoon, evening, and night reports at random times within their respective time blocks. Access to each of those reports expired 1 hour after the initial notification.

The participants could also complete a bedtime report, available from 8 PM to midnight, if they needed to go to sleep before receiving their prompt for the night report. These scheduled prompts assessed whether the participants had come across alcohol content in the media since their last submitted report. If the participants endorsed having any unreported alcohol content, they were asked whether it was one or multiple instances; for multiple exposures, they were instructed to think back on only the most recent instance of alcohol content when completing the subsequent set of questions. The participants who did not report encountering alcohol content were administered a set of general media questions that included self-reported media platform use and digital interactions unrelated to alcohol. This ensured that all the participants had the same opportunities to earn compensation based on survey compliance regardless of their daily alcohol content exposure.

Self-initiated media reports allowed youth to record exposure to media alcohol content in vivo. The participants initiated an assessment whenever they encountered an alcohol reference in the media. If the participants encountered >1 exposure in a time block, their first self-initiated media report fully assessed all alcohol media exposure content, including attitudes, cognitions, and context. Subsequent self-initiated media reports were abbreviated to reduce participant burden when submitting multiple exposures.

In all surveys, the participants were asked to share a screenshot, take a photo, type a text description, or include a link to a video. Images of alcohol content could be uploaded when submitting reports or taken within the app itself (videos uploads were also allowed). Youth were asked to describe the nature of the exposure, including the format or medium and source. They also indicated whether they reacted to the image (eg, like and comment). They also completed survey items assessing attitudes, cognitions (identification, liking, expectancies, and prototypes), and context related to the content. Abbreviated self-initiated media reports assessed only format or medium, source, and reaction. Measures and items are detailed in Table 1.

**Table 1:** Constructs assessed at the ecologic momentary assessment (EMA) report and weekly surveys.

	EMA (self-initiated or scheduled report)	Weekly
Exposure to alcohol-related content in media	Platform or medium Show, movie, or song name Sponsored advertisement or marketing Source of content Character presence	Frequency of exposure^a^ Platform or medium^a^
Engagement	Like, emoji, comment, etc Sharing of content	Like, emoji, comment, etc^a^
Identification with character	Want to be like the character, etc	—^b^
Prototypes (for content with characters)	Character is popular, attractive, cool, immature, etc	—
Context	Social context (alone or with others and in person or digitally interacting)	—
Exposure to other substance use content	Cannabis use^c^ Cigarette smoking^c^ e-Cigarette use^c^	—
Attitudes	I like or dislike content, disapprove or approve of content, etc	—
Perceived norms	Teenagers their age like or dislike content, disapprove or approve of content, etc	Descriptive norms (peer and close friend)^a^
Alcohol use	—	Alcohol use, subjective effects, and problems^d^ Intention and willingness to drink^a^ Alcohol available or offered^a^
Substance use	—	Cannabis use, cigarette smoking, and e-cigarette use Willingness to use cannabis or e-cigarettes^a^
Affect	—	Positive and negative affect^a^
Filler items	Open-ended text	Sleep^d^ Fun polls^a^

^a^Only assessed in the night or bedtime scheduled report.

^b^Not collected in this survey.

^c^Measured at the daily level via timeline follow-back.

^d^Measured at the weekly level.

#### Weekly Surveys

At the end of each week during the EMA protocol, the participants received a link to complete an online survey (on days 8, 15, and 22 of each burst). Items assessed past-week exposure to alcohol content, perceived peer and friend norms about alcohol use, and affect. This was followed by a 7-day TLFB assessment, which captured daily alcohol and other substance use over the past week.

#### Follow-Up Surveys

Follow-up surveys were administered 1 week before each burst. These surveys were identical to the baseline, except they excluded stable sociodemographic information (eg, sex assigned at birth and race or ethnicity).

#### Retention Methods

Study personnel contacted the participants daily via text or email and as needed to check progress, encourage compliance, and address technical issues. In addition, we created study social media accounts and encouraged the participants to follow them as a means to promote engagement and a sense of study community. Posts included the results of weekly polls as well as other fun content. We also communicated with the participants in between data collection bursts, including text check-ins, emailing certificates of burst completion, and mailing study swag, and we implemented a weekly survey raffle to encourage compliance with the weekly survey. A companion paper describing techniques for and data on retention and compliance is currently being prepared for submission.

### Ethical Considerations

#### Human Participant Protections

All procedures were approved by the Brown University institutional review board (protocol number 2009002813). Informed consent was obtained from the participants who were aged 18 years, and both parental consent and verbal assent was obtained for the participants who were younger than 18 years. Consenting materials were written at an 8th-grade reading level.

#### Methods to Maximize Confidentiality

We informed the participants during the orientation session that to protect data and prevent unauthorized access, study data were deidentified and stored in password-protected internal servers. We also encouraged the participants to ensure they enabled passwords to protect their phones and to exit out of the app and survey when complete. To protect against the risk that concerned parents might seek information from the study about their child, the consent and assent forms explained that we could not provide the parent with information that their child had given us.

#### Confidentiality of Image Content

Given that the participants could submit images that contained identifying information (eg, social media posts of friends), the participants were instructed to crop out usernames or faces and to cover or mark over faces and personal information. They were also told to delete screenshots or photos with identifying information from their phone and gallery after submitting the image.

#### Compensation

One of the keys to achieving high EMA response rates is designing compensation schedules that are linked to target response rates. The participants were coached to achieve a response rate of at least 3 reports (either prompted or self-initiated) per day. A complete day was defined as at least 1 report in 3 out of 4 time blocks (morning, afternoon, evening, and night or bedtime), and the participants were compensated US $5 per complete day. To encourage consistent responding, the participants could also earn a US $10 weekly bonus by having at least 6 complete days in a given week. In addition to EMA compensation, the participants could earn US $30 for completing both the baseline survey and the orientation session. They could also earn US $5 for each weekly survey completion and US $35 and US $40, respectively, for completing the first follow-up and second follow-up surveys. These incentives together meant that the participants could earn up to US $555 for participating. Level of compensation was commensurate to level of participant involvement and consistent with regional compensation norms for similar studies. All compensation was given in the form of Amazon e-gift cards. The compensation schedule was clearly delineated in the orientation materials and in the consent forms.

### Measures

#### Overview

Tables 1 and 2 present an overview of study measures. Across all survey types, skip patterns, error checks, and validation features were built in to ensure high-quality data and minimize data entry errors. Items were programmed in MetricWire, which is a commercially available app with features that are appropriate for smartphone-based experience sampling and EMA research. MetricWire also allows the user to take photographs or videos from within the app and permits uploads of screenshots or saved photos from a user’s phone.

**Table 2:** Constructs assessed in the baseline and follow-up surveys.

Measure	Subscales or sample items	Source
Sociodemographic and academic factors	Biological sex, gender, age, grade, racial and ethnic group, religious involvement, family household income, weight, and height Academic performance (grade point average, missed classes, and hours studying) Engagement	—^a^
Media use	Media ownership and use, intensity of use (h/d), how social media makes them feel about themselves and others, and how social media makes them feel about alcohol content	Social media and technology survey
Exposure to alcohol content in the media	Frequency of exposure to alcohol on each platform Who posts alcohol content Types of engagement with alcohol content Personal alcohol posting behavior Exposure to alcohol advertising	Pew Research Center [48], Wartella et al [49], and Roberts et al [50]
Media literacy	Like content Critical thinking about message source Critical thinking about message content	Austin et al [51]
Online social identity	Whether being a member of one’s online social network is an important reflection of who they are	Pegg et al [52]
Parental monitoring of digital use	Parental controls, check social media profiles and websites visited, take away privileges Parents have talked with them about appropriate: online behavior toward others; content to share online; content to be viewing online; and content to be viewing, hearing, and reading in the media	Anderson [53]
Alcohol use	Frequency of drinking and heavy drinking, typical number of drinks, subjective effects (high, drunk), availability of alcohol, age of initiation, first sip context, and shortened Rutgers Alcohol Problems Index	Adapted from National Institute on Alcohol Abuse and Alcoholism; Dawson [54], Rehm [55] and White and Labouvie [56]
Other substance use	Frequency and types of cannabis, cigarettes, e-cigarettes, tobacco dependence, classes of illicit drugs, and (nonmedical) prescription drug use Age of initiation for all substances used	Adapted from the National Youth Tobacco Survey [57] and Adolescent Brain Cognitive Development Study [58] and items adapted from Morean et al [59]
Peer norms	Injunctive norms of close friends Descriptive norms close friends and kids your age	Peer Passive Social Influence questionnaire and Wood et al [60,61]
Deviant peers	My friends are very smart My friends get into trouble with the police	Social development scale [58]
Peer orientation	Willingness to sacrifice to be popular and have friends	Peer Orientation Scale [62]
Parental monitoring	Knowledge of child activities (eg, Do your parents know what you do during your free time?)	9-item Parental Monitoring Questionnaire [63]
Affect	Positive and negative affect	Positive and Negative Affect Schedule [64]
Anxiety	Social anxiety	Adolescent Version of Social Anxiety Scale for Children [65]
Stress	In the past month, please describe how often have you...felt that you were on top of things?	Perceived Stress Scale [66]
Life events	Family moved; parents got divorced	Adverse Life Events and Resilience [67]
Impulsive behavior	Premeditation, sensation seeking, perseverance, and urgency (positive and negative)	UPPS-P Impulsive Behavior Scale [68]
Need for cognition	I find satisfaction in deliberating hard and for long hours	Need for Cognition scale [69]
Motivation to seek out or approach fun activities	I will often do things for no other reason than they might be fun	Behavioral Activation fun-seeking subscale [70]
Leisure boredom scale	Leisure experience (awareness, boredom, challenge, and distress)	Leisure Boredom Scale [71]
Alcohol expectancies	Affective, cognitive, and behavioral effects of alcohol use were assessed following the prompt: “How likely is it that the following would happen to someone your age if they had one or more alcoholic drinks?	Schell et al [72] and adapted from the Alcohol Expectancy Questionnaire—Adolescent
Drinking identity	How much drinking plays a part in one’s life (eg, drinking is a part of who I am)	Alcohol Self-Concept Scale [73]
Drinker prototypes	How much does the word “POPULAR” describe your image of a typical high school student who chooses not to drink alcohol?	Adapted from a measure developed by Gerrard et al [74]
Explicit alcohol approach	I would have liked to have a drink or two	Inclined/Indulgent subscale of Approach and Avoidance of Alcohol Questionnaire [75]
Family history of alcohol problems	Did you ever wish that a parent would stop drinking?	6-item Children of Alcoholics Screening Test [76]

^a^Not applicable.

#### EMA Measures

For all reports of alcohol content in the media, the report first assessed the format or medium (all that apply): Snapchat, Instagram, TikTok, Facebook, YouTube, movie, TV show, music (eg, radio and Spotify), or other. For social media (Snapchat, Instagram, TikTok, and Facebook), we assessed who posted or shared the content, with the following response options: “sponsored ad/commercial that appeared,” “company/brand I follow,” “influencer/celebrity I follow,” “friend or similar-aged family member I know well,” “older friend or family member I know well,” and “someone I know but not well.” We also asked whether the content was posted publicly or privately and about who created the content, with the following response options: “same person who shared it,” “company/brand I follow,” “influencer/celebrity I follow,” “friend or similar-aged family member I know well,” “older friend or family member I know well,” “someone I know but not well,” and “someone I do not know/not sure.” Participant engagement with the content was assessed, including “liked,” “other reaction (eg, emoji),” “commented,” “shared/posted publicly,” “shared/posted privately,” “saved it or added it to a list,” “followed user/account or related group,” “blocked or deleted the user/account,” “clicked on an affiliated link,” “discussed with someone else, in person,” and “nothing (I kept scrolling).” If “shared/posted privately” was endorsed, we asked who they shared it with; response options were “close friend/best friend,” “significant other (eg, boyfriend or girlfriend),” “friend/acquaintance,” “peer (eg, classmate or coworker),” “adult family member,” “sibling/cousin similar in age,” and “other.”

For music, we asked the participants the song name and artist in a text field. For YouTube videos, we asked the participants to enter the link (URL). We also asked whether the alcohol content was part of the YouTube video or in a sponsored ad/commercial that appeared before/during the video. Likewise, for movies and TV shows, we asked whether the alcohol content was part of the movie or show or in a sponsored ad/commercial that appeared. We also asked the participants to enter the name of the movie or show and time stamp (if known) in a text field.

For all types of content, we assessed whether there were people shown or described in the alcohol content. If there were multiple people, we asked the participant to think of the person who was most engaged with alcohol. If there were people shown, we asked who the person was with the following response options: “friend/peer,” “celebrity/influencer,” “somebody I did not know,” and “other.” We asked the participants to evaluate the person along 4 domains: I want to be like this person, I wish I could do what they do, I wish I could have as much fun as they have, and I felt like the person was similar to me; each item was rated on a scale from 1 (strongly disagree) to 7 (strongly agree). The participants rated their perceptions of the person’s or character’s (1) popularity, (2) attractiveness, (3) coolness, (4) immaturity, (5) confusion, and (6) self-centeredness (prototypes) [74] using a scale from 0 (not at all) to 3 (very). The participants reported on their own attitudes as well as beliefs about teenagers their age (perceived norms) with respect to the following domains: (1) content promotes or discourages alcohol drinking, (2) liking or disliking content, (3) disapproval or approval of the content, (4) content makes drinking seem better or worse than it really is, and (5) more or less likely to drink upon viewing content (response options: better, neutral, and worse). We also asked whether the creators of the content were intending to promote or discourage drinking.

We asked respondents whether they were with someone or others (in person) when they encountered the content and, if so, who it was: “alone,” “with a close friend or best friend,” “with a significant other (eg, boyfriend or girlfriend),” “with a friend or acquaintance,” “with a peer (classmate or coworker),” “with an adult family member,” and “with a sibling or cousin similar in age.” We also asked how many other people the respondent was digitally interacting with (such as texting, online messaging, video chat, etc) when they encountered the content and, if so, how many people (none, 1, 2 or 3, and ≥4). Finally, we asked whether the participant came across any examples of marijuana (weed and cannabis), e-cigarette use, or cigarette smoking that day (bedtime report only).

In total, the self-media report contained 32 items. The scheduled media reports contained 32 items if the participant endorsed encountering alcohol content. However, participants were shown only a subset of these items due to planned skip patterns (eg, if the participant endorsed YouTube, they were not shown the 6 items about other media types, and if the participant noted there were no people shown or described in the content, they were not shown the 3 items specific to people). The shortened versions of self-media reports contained 13 items, although again participants were only shown a subset of these items. If the participant did not endorse encountering alcohol content in the scheduled media report, they completed 6 items. Consequently, most reports took between 1 and 3 minutes.

#### Content Coding

Coding manuals were developed for both image and text submissions. We strove to make the coding systems similar to one another, as the participants were given the choice of whether to upload an image or to enter text for each digital media type (eg, taking a screenshot of a song or lyrics on Spotify and entering the name of the song or relevant lyrics into the text box). The participants were also provided an open-ended text box at the end of the survey where additional information could be entered (eg, describing a scene in a film for which a screenshot was provided). For the image coding, student coders underwent a rigorous training process to ensure interrater reliability and consensus. This involved 2 rounds of training with 10 coders (80 images) each. Interrater reliability of the training images was >90%. Codes with lower reliability (eg, social and sophistication) were redefined using advisory board input, and trainers were retrained. A companion paper describing the content coding process is currently being prepared for submission.

#### Weekly Measures

A 7-day TLFB assessment queried alcohol use, cigarette use, e-cigarette use, cannabis use, and sleep for each day, using an online TLFB [77]. We also assessed whether the participants experienced any of the following alcohol-related problems, which were a subset of items from the Rutgers Alcohol Problem Index [56]: hangover, nausea or vomiting, self-injury, driving after drinking, blackout, rude or aggressive behavior, and unwanted sex. Alcohol use and related consequences were assessed on a weekly rather than daily basis to separate the assessment of media alcohol content from the assessment of drinking behavior.

We assessed whether the participant had planned or intended to drink but did not, whether they were at a place where alcohol was available, and whether they were offered alcohol in the past week. We assessed willingness to drink alcohol, use marijuana or weed, and use an e-cigarette if offered by a friend or classmate using the following prompt: *“*If a friend or classmate offered you [substance], would you use it?” Response options ranged from 0 (definitely not) to 3 (definitely yes) [78]. We assessed descriptive norms for close friends (“How often do you think that your close friends drank alcohol in the past week?”) and peers (“How often do you think that the typical student in your grade drank alcohol in the past week?”); response options ranged from 0 (did not drink alcohol) to 4 (drank alcohol daily or nearly daily), adapted from a measure used by Wood et al [60] to reflect past-week use. Injunctive norms were assessed by asking how most of their close friends feel about kids drinking alcohol, getting drunk, using marijuana or weed, or using e-cigarettes; responses ranged from 1 (strongly disapprove) to 5 (strongly approve) [60]. We also assessed positive and negative affect (5 items each) using the Positive and Negative Affect Schedule [64]; responses ranged from 1 (very slightly or not at all) to 5 (extremely).

In addition, the participants reported whether they had seen any alcohol-related content in the past week (and if so, the media platform and whether they engaged with it), as well whether they came across any content that specifically referred to positive and negative consequences of drinking. Finally, we asked whether they had publicly or privately posted any text, images, or videos of alcohol in the past week.

#### Baseline and Follow-Up Survey Measures

At baseline and preceding each burst, a web-based survey assessed sociodemographics, media, alcohol consumption and related problems, other substance use, drinking context, and source of alcohol (Table 2 provides a list of all measures).

### Data Management and Analysis Plan

The scheduled and self-initiated media reports were cleaned and merged by participant ID into a single database with all daily media reports. Coded image and text descriptions of media will be merged into the database when all coding is complete. The weekly survey provides variables for alcohol use at the level of the day (which can be merged with EMA data aggregated at the level of the day), at the level of the week (aggregated across 7 days), and at the level of the burst (aggregated across 21 days).

Models will include sex, age, and seasonality (dummy coded, summer as reference group) as controls. We will examine bivariate associations and test basic mean differences across subgroups and time with generalized linear models with the appropriate link function. Given concerns about spurious findings and replicability, primary analyses were planned a priori; secondary analytic analysis will be thoughtfully planned and based on a priori hypotheses. We will preregister analytic plans and hypotheses. We will control type 1 error by distinguishing primary and secondary measures, testing a priori hypothesized associations, and using ensemble-type tests.

To document adherence to the protocol, we will compute the proportion of reports completed overall, consistent with the broader literature [34,35]. We will also compute completion rates for each survey type (eg, morning and evening) and for each burst to identify trends (declines) in average compliance rates as the monitoring period progresses. We will calculate the proportion of reports completed each day and compute the number of participants who complete at least 1 report on a given day (which we anticipate will be high) as well as complete all reports in a day (which we expect to be moderate). We will also examine adherence with surveys required for compensation (≥3 completed surveys per day). Finally, we will examine the number of items completed and the amount of time (in min) from prompt signal to answering of prompt, consistent with recommendations in the field [79]. Inattention to survey content may be reflected by incomplete surveys or surveys completed excessively fast. We will examine correlates of noncompliance as a function of person-level characteristics (eg, gender and experience consuming alcohol) and within-person survey or day-level characteristics (eg, time of day [80]). We will test for a fatigue effect in reported exposure; if necessary, we will correct for this using inverse probability weights [31].

To quantify and characterize in vivo exposure to media alcohol content, proportions and means (SDs) will be computed to describe exposure frequency, duration, and format or medium. We will test group differences using parametric (correlations and regressions or general linear models) and nonparametric (chi-square) tests. We can also conduct latent class analysis to characterize different domains of media exposure. To examine differences between constructs at times of exposure versus nonexposure, we will compare event-level data versus random prompt data using multilevel models (MLMs), which account for clustering (events within person) and permit varying numbers of observations. Level 1 (L1) corresponds to within-person effects, including event-level and random assessments. Level 2 (L2) corresponds to between-person effects (eg, sex or L1 variables aggregated over time). L1 variables will be person centered, and L2 variables will be grand mean centered. Compositional effects are tested by aggregating the L1 variable. We will model bursts as a time-varying effect, as there are too few for a 3-level MLM.

To examine prospective associations between media alcohol content exposure and alcohol use, we will conduct MLM for short-term time frames. We will conduct cross-lagged panel models for longer-term time frames, including a 1-week time frame and a 4-month time frame. In the cross-lagged models, psychometric work will establish reliable latent variables for media exposure and alcohol involvement. We will control for key social influences on adolescent drinking, including close friend and peer norms about alcohol use and familial influences (eg, parental monitoring, parental communication and rules about alcohol use, and parental alcohol use), as assessed in weekly surveys and baseline and follow-up surveys.

To identify mechanisms of the association between media alcohol content exposure and drinking, we can test mediation by the putative mechanisms, predicting alcohol use at the burst level from event-level exposure. Mediation of event-level media exposure by an event-level mediator (eg, identification with the character) can be modeled. Alternately, mediation could be tested at the burst level, for example, by aggregating media content across the 21 days and using a mediator from a (preceding) follow-up survey. We will also control for relevant covariates, particularly when examining event-level prototypes and week-level social norms.

Analytic models will handle missing data under assumptions that data are missing at random using maximum likelihood estimation [81,82], including full information maximum likelihood, which is available in current software packages such as Mplus [83]. We will incorporate correlates of missingness to provide information for our model based on our findings from our examination of both person- and event-level correlates of noncompliance.

## Results

This research received funding in September 2020. Following a qualitative phase of data collection, EMA data collection took place from February 2022 to August 2023. Data management is ongoing. Descriptive preliminary analyses are underway; however, hypothesis testing for the study aims has not yet begun. Preliminary data have been disseminated through conference presentations in 2024-2025 and manuscripts are currently ongoing with full results anticipated to be published in 2025 and 2026.

## Discussion

### Overview

This paper describes a fine-grained ecological protocol that addresses the fundamental question of how media alcohol content elevates underage drinking risk to optimally inform next steps in preventive media literacy intervention research. Existing media intervention programs are less equipped to handle new media, focusing on those in middle childhood and failing to engage adolescents (a group arguably at greatest need for intervention), and they do not target media as it is experienced in vivo on portable devices. By characterizing adolescents’ real-world, in vivo exposure to alcohol content in the media, the study will generate essential insights to inform the development and implementation of digital interventions tailored for delivery via mobile devices.

### Anticipated Findings

By querying exposure to alcohol content throughout the day, study findings will inform the nature of alcohol content, including platform (social media, YouTube, film, television, and music), source, and engagement. We expect to observe high rates of alcohol exposure in entertainment media and YouTube but anticipate that the degree of exposure in social media will vary as a function of source (eg, less alcohol content will be observed when the content is posted by same-age peers or friends relative to content posted by the alcohol industry or influencers). We also anticipate greater engagement when content is posted by peers or friends. Ancillary data from content-coded images and text descriptions will provide a highly rich representation of media content. At the same time, content coding of these data may prove to be challenging to do by hand and may necessitate artificial intelligence and machine learning approaches to fully capitalize on the data captured.

The study will assess youth appraisal of media content as it is encountered and thereby pinpoint momentary factors that may account for media’s influence on drinking behavior. Cognitive mechanisms (expectancies and prototypes) are expected to vary as a function of exposure at a more proximal, momentary scale (and hence provide superior digital intervention targets) as compared to social mechanisms, which may require a cumulative or distal influence. Given the inherent limitations of conducting research in a constantly evolving area, such as entertainment and social media, there is value in shifting focus to the underlying mechanisms or processes (eg, targeting beliefs about alcohol content on a given platform rather than targeting time spent on that platform, given that the platform may change or be replaced over time). We attempted to keep abreast of recent advances in social media (eg, incorporating BeReal midway through the study), but it was difficult to do so in a systematic way.

The multiburst design permits testing of prospective bidirectional associations between exposure to media alcohol content and alcohol involvement across short- and longer-term time frames. Longitudinal data also will be used to test whether effects of exposure on alcohol cognitions, norms, and use are cumulative over platform and over time, consistent with the idea of a reinforcing effect of repeated exposure. The most effective approach to mitigating the influence of the media is to break the reciprocal cycle of media exposure leading to drinking, and vice versa, by reducing both the effects of media exposure on adolescent drinking and the extent to which at-risk adolescents seek out media content that normalizes and glamorizes alcohol use. Targeting constructs that are induced by in vivo media exposure at the time of exposure may be a highly effective strategy for breaking this cycle.

### Next Steps

Although not described here, next steps are to conduct focus groups with a subset of the participants who completed the study to understand youth’s lived experience of in vivo exposure to media alcohol content and to obtain their perceptions about the content, the effects of media exposure on them and their peers, and their reactions to proposed intervention targets. Both this study and the subsequent qualitative work will launch a program of research to counter the effects of alcohol-related media exposure as it is experienced in vivo by adolescents in an effort to minimize underage alcohol involvement.

## Abbreviations

EMA: ecological momentary assessment
MLM: multilevel model
RA: research assistant
TLFB: timeline follow-back
YAB: youth advisory board

## Data Availability

Data will be made available through the National Institute on Alcohol Abuse and Alcoholism Data Archive, a data repository housed within the National Institute of Mental Health Data Archive.
